# Palmitoylation-driven immune dysregulation and prognostic signature in low-grade glioma: a multi-omics and functional validation study

**DOI:** 10.3389/fphar.2025.1586921

**Published:** 2025-06-04

**Authors:** Zehao Wang, Tianlun Yu, Yuqiao Liu, Yufan Wu, Jingqing Hu

**Affiliations:** ^1^ School of Basic Medical Sciences, Chengdu University of Traditional Chinese Medicine, Chengdu, Sichuan, China; ^2^ Clinical Laboratory, Kunshan Rehabilitation Hospital, Zhou Shi Branch, Kunshan Traditional Chinese Medicine Hospital, Kunshan, China; ^3^ Science and Technology Office, Kunshan Hospital of Traditional Chinese Medicine, Kunshan, China; ^4^ School of Traditional Chinese Medicine, Tianjin University of Traditional Chinese Medicine, Tianjin, China

**Keywords:** palmitoylation, LGG, prognostic signature, tumor microenvironment, immune infiltration, IGFBP2, immunotherapy

## Abstract

**Background:**

Palmitoylation, a critical post-translational modification, regulates protein localization and function in cancer. However, its role in glioma progression, immune modulation, and prognosis remains poorly understood.

**Methods:**

We integrated transcriptomic, clinical, and mutation data from multicenter cohorts to analyze 30 palmitoylation-related genes in low-grade gliomas (LGG). Consensus clustering, differential expression analysis, and LASSO regression were employed to define palmitoylation clusters, identify prognostic genes, and construct a risk signature. The evaluation of immune infiltration and immunotherapy efficacy was further conducted across different risk groups. In the palmitoylation-related risk model, IGFBP2 was functionally validated through siRNA-mediated knockdown and a series of assays, including EdU incorporation, cell cycle analysis, wound healing, and transwell migration assays.

**Results:**

Two palmitoylation clusters (A/B) were identified, with Cluster B exhibiting poorer survival (*P* < 0.001), enriched JAK-STAT signaling, and elevated immune infiltration (M1/M2 macrophages, CD8^+^ T cells). A five-gene prognostic signature (*CHI3L1*, *IGFBP2*, *MEOX2*, *EMILIN3*, *SFRP2*) demonstrated robust predictive accuracy in training (AUC 0.92–0.94) and validation cohorts (AUC 0.68–0.83). High-risk patients showed upregulated PD-1, PD-L1, and CTLA4 (*P* < 0.001) and higher TIDE scores, indicative of immune dysfunction. IGFBP2 knockdown suppressed glioma cell proliferation (*P* < 0.01) and migration (*P* < 0.001), linking it to tumor aggressiveness.

**Conclusion:**

Palmitoylation plays a pivotal role in LGG progression by influencing immune evasion and stromal interactions. The developed prognostic signature and nomogram offer practical tools for risk stratification in clinical settings, with IGFBP2 identified as a promising therapeutic target. These insights highlight the potential of palmitoylation-focused therapies to enhance outcomes for LGG patients.

## Highlights

• Identified two palmitoylation clusters with distinct survival (Cluster B: HR = 2.3, p < 0.0001).

• Developed a five-gene prognostic model (AUC up to 0.94) validated across TCGA and CGGA cohorts.

• High-risk patients exhibit immune-excluded phenotypes with elevated PD-L1/CTLA4 and TIDE scores.

• IGFBP2 silencing reduced LGG proliferation and migration (p < 0.0001).

## 1 Methods

### 1.1 Data acquisition

We retrieved gene expression data and clinical details for low-grade glioma (LGG) patients from the Cancer Genome Atlas (TCGA) and the Chinese Glioma Genome Atlas (CGGA). Only pathologically confirmed WHO grade II/III diffuse gliomas were included. Patients with prior radiotherapy/chemotherapy or other neurological disorders were excluded. The expression data of GTEx normal tissues included in the Xena database were standardized using the Toil process. FPKM values were converted to TPM using the formula: TPM = (FPKM/ΣFPKM) × 10^6^. Batch effects between TCGA and CGGA datasets were corrected using the ComBat algorithm to ensure data consistency. Mutation and Copy Number data were collected from the cBioPortal website, encompassing 652 patients/680 samples from various studies: Brain Lower Grade Glioma (TCGA, PanCancer Atlas), Low-Grade Gliomas (UCSF, Science 2014), Anaplastic Oligodendroglioma, Anaplastic Oligoastrocytoma (MSK, Neuro Oncol 2017), and Pilocytic Astrocytoma (ICGC, Nature Genetics 2013). In this study, we utilized TCGA data as the training set, while two cohorts from CGGA were employed as the validation set. The list of palmitoylation regulating genes (ZDHHC1, ZDHHC2, ZDHHC3, ZDHHC4, ZDHHC5, ZDHHC6, ZDHHC7, ZDHHC8, ZDHHC9, ZDHHC11, ZDHHC12, ZDHHC13, ZDHHC14, ZDHHC15, ZDHHC16, ZDHHC17, ZDHHC18, ZDHHC19, ZDHHC20, ZDHHC21, ZDHHC22, ZDHHC23, ZDHHC24, LYPLA1, LYPLA2, ABHD17A, ABHD17B, ABHD17C, PPT1, PPT2) was derived from published literature (Cai et al., 2023; Qian et al., 2020).

### 1.2 Consensus clustering analysis of palmitoylation genes and Principal Component Analysis (PCA)

The common prognosis-related palmitoylation genes identified through Cox regression analysis were utilized for subsequent unsupervised cluster analysis. We divided the samples into Palmitoylation Clusters A and B, testing k values from one to nine. We selected the k value with the best clustering stability, characterized by lower coefficient of variation, higher clustering consistency, and a relatively steep cumulative distribution function (CDF) curve. The classification of LGG patients into two clusters was further validated by Principal Component Analysis (PCA) performed with the “stats” package.

### 1.3 Gene Set Variation Analysis and single-sample gene set enrichment analysis (GSVA and ssGSEA)

GSVA is a powerful tool for analyzing gene sets and identifying pathway enrichment. It is an unsupervised and non-parametric method for scoring gene sets and converting them to pathway levels. For enrichment analysis, we downloaded the “c2. cp.kegg.v7.2. symbols.gmt” gene set from MSigDB, a comprehensive collection of annotated gene sets. We used the GSVA algorithm to calculate the scores for each gene set and explore the biological functional differences between the two Palmitoylation clusters. We set the threshold for significant enrichment at adjusted P < 0.05. The ssGSEA algorithm, based on immune gene sets, considered genes related to various immune cell types, pathways, functions, and checkpoints. In this study, we used the “GSVA” R package to comprehensively evaluate the immune landscape of each LGG sample in different Palmitoylation clusters (Cao et al., 2023).

### 1.4 Identifying and clustering of DEGs between the palmitoylation clusters

The “limma” R package was employed to detect differentially expressed genes (DEGs) between the two palmitoylation clusters, applying thresholds of |logFC| > 1 and adjusted P < 0.001. Functional annotation through GO analysis examined biological processes, cellular components, and molecular functions, while KEGG enrichment analysis revealed significantly enriched signaling and immune-related pathways within the DEGs of the palmitoylation clusters. The “ConsensusClusterPlus” R package was then used to perform clustering analysis on these genes, following previously established methods for palmitoylation genes.

### 1.5 Palmitoylation-related prognostic signature construction

Based on the 1,543 DEGs from TCGA training set, we identified 1,081 prognosis-related genes that were common to both the CGGA325 and CGGA693 cohorts through univariate Cox regression analysis. A prognostic signature was constructed using the LASSO algorithm implemented in the “glmnet” R package. Ten-fold cross-validation (nfold = 10) was performed to determine the optimal lambda value, which was selected as the value minimizing the cross-validation error (cvfit$lambda.min). The robustness of the prognostic signature was confirmed through external validation sets (CGGA325 and CGGA693). Samples were stratified into high-risk and low-risk categories using the median risk score derived from the training set. The predictive ability of the signature was evaluated using KM survival analysis and ROC curves in the training and validation sets using the “timeROC” R packages. Additionally, we created a nomogram using the “rms” R packages to predict 1-, 3-, and 5-year survival rates and calibrated the model to assess its consistency with practice.

### 1.6 Immune microenvironment and immunotherapy assessment

The ssGSEA algorithm was also used to assess the diverse immune cell infiltrations, and the “estimate” R package was employed for estimating the tumor microenvironment (TME), encompassing stromal score, immune score and estimate score. Tumor Immune Dysfunction and Exclusion (TIDE) (http://tide.dfci.harvard.edu) serves as an analytical instrument for assessing the likelihood of tumor immune evasion. We standardized the mean value of each gene across all samples, as stipulated by the website.

### 1.7 siRNA treatment

The U251 low-grade glioma (LGG) cell line was acquired from the American Type Culture Collection (Manassas, VA, United States). Three IGFBP2 siRNAs were purchased from GenePharma (Shanghai, China) and transfected into U251 cells using InvitroRNA™ (InvivoGene Biotechnology, Suzhou, China). Non-targeting siRNAs (GenePharma, Shanghai, China) were used as negative controls in the experiments.

### 1.8 qRT-PCR

To analyze IGFBP2 gene expression and its alterations following siRNA transfection, qRT-PCR was conducted. RNA was isolated from U251 cells using TRIzol reagent (Takara, Japan), followed by cDNA synthesis with HiScript II qRT SuperMix (Vazyme, China). The qRT-PCR assay was subsequently carried out using ChamQ Universal SYBR qPCR Master Mix (Vazyme, China). The primer sequences used for qRT-PCR are as follows:

IGFBP2:

Forward: 5′-GCA​CTT​GTG​AGA​AGC​GCC​G-3′

Reverse: 5′-GCC​TCC​TTC​TGA​GTG​GTC​ATC-3′

GAPDH:

Forward: 5′-TGC​ACC​ACC​AAC​TGC​TTA​GC-3′

Reverse: 5′-GGC​ATG​GAC​TGT​GGT​CAT​GAG-3′

### 1.9 Western blot analysis

Proteins were extracted from U251 cells using RIPA lysis buffer (Beyotime, China) containing a protease inhibitor cocktail (Roche, Switzerland). Protein concentrations were measured using a BCA assay (Thermo Fisher, United States), and 30 µg of each sample was resolved on 10% SDS-PAGE gels before being transferred to PVDF membranes (Millipore, United States). The membranes were blocked with 5% non-fat milk in TBST for 1 h at room temperature and then probed with primary antibodies overnight at 4°C: anti-IGFBP2 (1:1,000, Abcam, ab123456) and anti-β-actin (1:5,000, Proteintech, 66009-1-Ig). After TBST washes, the membranes were incubated with HRP-conjugated secondary antibodies (1:5,000, Cell Signaling Technology, 7074S) for 1 h at room temperature. Protein bands were detected using an ECL system (Tanon, China) and captured with a ChemiDoc™ XRS + System (Bio-Rad, United States).

### 1.10 EdU incorporation assay

Cell proliferation was evaluated using the EdU Cell Proliferation Kit (BeyoClick™, China). U251 cells were plated in 24-well plates and treated with 10 μM EdU for 2 h at 37°C. After fixation with 4% paraformaldehyde for 15 min and permeabilization with 0.3% Triton X-100 for 20 min, cells were stained with Click Reaction Mix (containing Alexa Fluor 594) for 30 min in the dark. Nuclei were labeled with Hoechst 33,342 (Beyotime, China) for 10 min. Fluorescent images were acquired using a Nikon Eclipse Ti2 microscope (Japan), and EdU-positive cells were analyzed using ImageJ software (NIH, United States).

### 1.11 Cell cycle analysis by flow cytometry

Cells were collected, fixed in 70% ethanol overnight at 4°C, and incubated with 50 μg/mL propidium iodide (PI; Sigma, United States) and 100 μg/mL RNase A (Beyotime, China) for 30 min at 37°C in the dark. Cell cycle phases (G0/G1, S, and G2/M) were assessed using a CytoFLEX flow cytometer (Beckman Coulter, United States) and analyzed with CytExpert software (v2.4), with quantification based on DNA content.

### 1.12 Apoptosis detection by flow cytometry

Apoptosis was assessed using the Annexin V-FITC/PI Apoptosis Detection Kit (BD Biosciences, United States). Cells were harvested, washed with PBS, and suspended in 100 μL binding buffer. After adding 5 μL Annexin V-FITC and 5 μL PI, cells were incubated for 15 min at room temperature in the dark. Apoptotic cells were measured using a BD FACSCanto II flow cytometer (BD Biosciences, United States), and data were analyzed with FlowJo software (v10.8), distinguishing early apoptotic (Annexin V+/PI−) and late apoptotic (Annexin V+/PI+) populations.

### 1.13 Wound healing assay and transwell migration assay

Twenty-four hours post-transfection with IGFBP2 siRNA, U251 cells were plated in 6-well plates, and a wound was introduced using a 1-mL pipette tip. Cell migration was monitored by capturing images at specific time intervals. For the transwell migration assay, U251 cells were placed in the upper chamber of transwell inserts with 200 µL of serum-free medium, while the lower chamber contained 500 µL of medium supplemented with 20% fetal bovine serum. After 24 h of incubation at 37°C, cells that migrated to the lower chamber were fixed with 4% paraformaldehyde, stained with 0.1% crystal violet, and visualized under an optical microscope. Migrated cells were quantified and documented.

### 1.14 Statistical analyses

All experiments were independently repeated three times. Data are presented as mean ± SD. Comparisons between two groups were conducted using t-tests for normally distributed data and Wilcoxon rank-sum tests for non-normally distributed data. For multiple group comparisons, analysis of variance (ANOVA) was applied for parametric data, while the Kruskal–Wallis test was used for non-parametric data. Survival analysis was performed using the Kaplan-Meier method, with statistical significance set at P < 0.05. All analyses were carried out using R software (version 4.2.0) and GraphPad Prism software (version 7.0).

## 2 Introduction

Glioma, the most prevalent primary malignant tumor of the central nervous system, continues to pose significant therapeutic challenges due to its aggressive progression, immunosuppressive microenvironment, and resistance to conventional therapies (Varn et al., 2022). Despite advances in molecular stratification, the prognosis for patients remains dismal, underscoring the urgent need for novel biomarkers and therapeutic strategies (Alessio et al., 2022). Post-translational modifications (PTMs), such as palmitoylation, have emerged as critical regulators of oncogenic signaling and immune evasion in cancers. This reversible lipid modification, mediated by a family of ZDHHC enzymes and depalmitoylases (e.g., LYPLA, PPT), governs protein localization, stability, and interaction networks (Pan and Chen, 2022). While palmitoylation dysregulation has been implicated in malignancies like breast and lung cancer, its role in shaping LGG progression, immune modulation, and clinical outcomes remains poorly characterized.

Recent studies suggest that palmitoylation may influence tumor-immune crosstalk by modifying immune checkpoint proteins or chemokine receptors (Yao et al., 2019). However, a systematic exploration of palmitoylation-related genes (PRGs) in LGG heterogeneity, prognostic stratification, and therapeutic vulnerability is lacking. Moreover, the interplay between Palmitoylation-related DEG subtypes, immune infiltration patterns, and immunotherapy response remains unexplored. Addressing these gaps could unravel mechanisms underlying LGG immune evasion and identify actionable targets to improve patient outcomes.

In this study, we integrated multi-omics data from The Cancer Genome Atlas (TCGA) and Chinese Glioma Genome Atlas (CGGA) cohorts to delineate palmitoylation-related molecular subtypes and their clinical implications (Roychowdhury and Chinnaiyan, 2016; Qian et al., 2021). Through consensus clustering and functional enrichment analyses, we identified two distinct palmitoylation clusters with divergent survival outcomes and immune landscapes. Cluster B, characterized by elevated immune infiltration and enriched JAK-STAT signaling, paradoxically exhibited poorer prognosis, suggesting an immunosuppressive microenvironment despite abundant immune cell presence. Leveraging differentially expressed genes (DEGs) between clusters, we constructed a robust five-gene prognostic signature (CHI3L1, IGFBP2, MEOX2, EMILIN3, SFRP2) validated across independent cohorts (AUC: 0.68–0.94). High-risk patients demonstrated upregulated immune checkpoints (PD-1, PD-L1, CTLA4) and elevated Tumor Immune Dysfunction and Exclusion (TIDE) scores, indicative of immunotherapy resistance. Functional validation revealed that IGFBP2 silencing suppressed LGG cell proliferation, migration and invasion, positioning it as a potential therapeutic target.

The study highlights the dual impact of palmitoylation in promoting LGG progression and facilitating immune evasion, establishing a prognostic framework for risk assessment and supporting the development of palmitoylation-targeted therapies to address treatment resistance. By addressing key gaps in the understanding of the PTM-immune axis in LGG, this research provides valuable translational insights for advancing personalized oncology.

## 3 Results

### 3.1 Palmitoylation clusters in LGG

**FIGURE 1:**
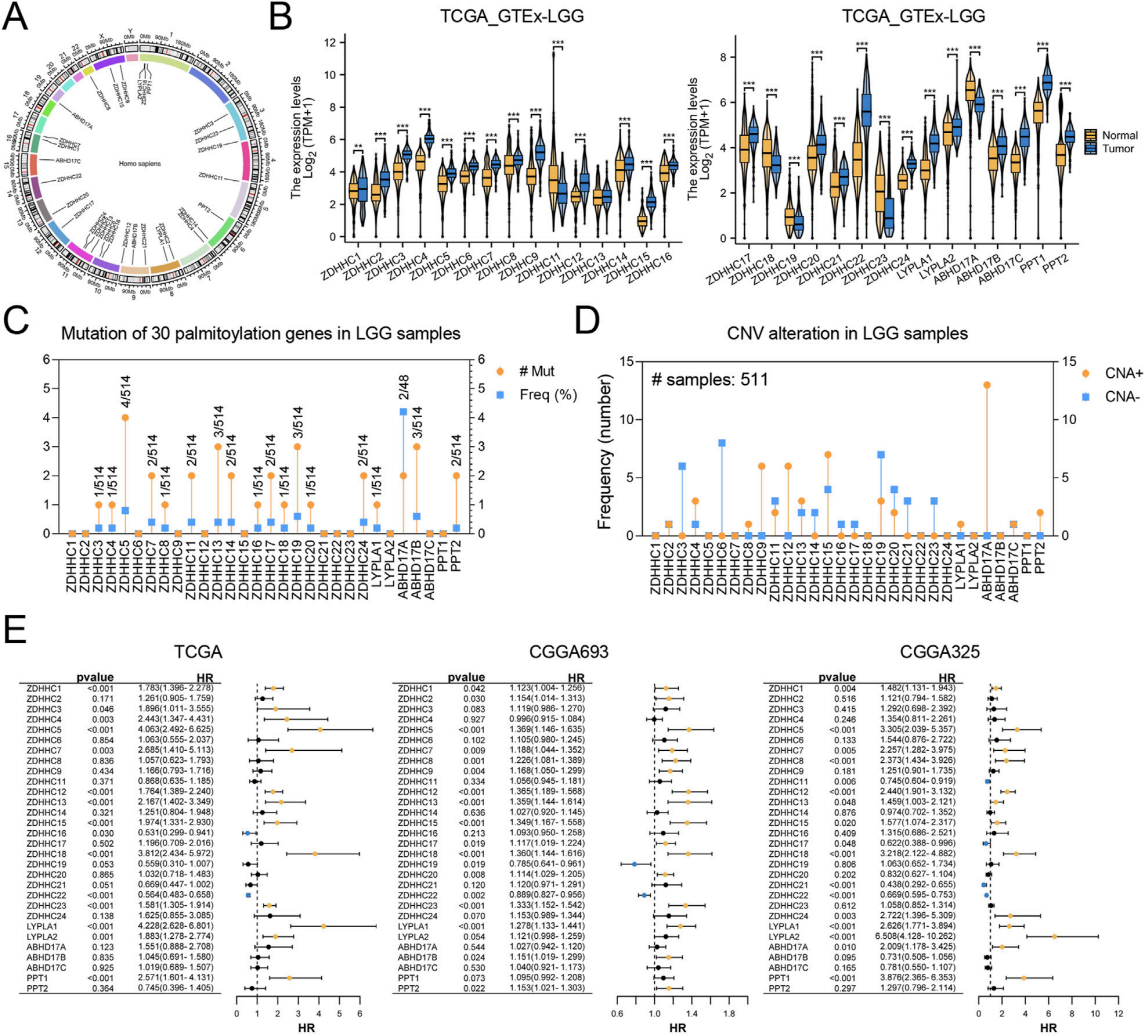
Analysis of Palmitoylation Regulating Genes in LGG. **(A)** Chromosomal localization of 30 palmitoylation regulating genes. **(B)** Expression levels of palmitoylation regulating genes in TCGA-LGG and GTEx normal tissues. **(C)** Mutation frequencies of palmitoylation regulating genes in LGG patients using the cBioPortal database. **(D)** Copy number variations (CNVs) of palmitoylation regulating genes in LGG patients. **(E)** Forest plot of univariate Cox regression analysis for the association between palmitoylation regulating genes and prognosis in CGGA_325, CGGA_693, and TCGA datasets.

In this study, we focused on the analysis of 30 palmitoylation regulating genes (ZDHHC1, ZDHHC2, ZDHHC3, ZDHHC4, ZDHHC5, ZDHHC6, ZDHHC7, ZDHHC8, ZDHHC9, ZDHHC11, ZDHHC12, ZDHHC13, ZDHHC14, ZDHHC15, ZDHHC16, ZDHHC17, ZDHHC18, ZDHHC19, ZDHHC20, ZDHHC21, ZDHHC22, ZDHHC23, ZDHHC24, LYPLA1, LYPLA2, ABHD17A, ABHD17B, ABHD17C, PPT1, PPT2). First, we analyzed the chromosomal localization of these 30 genes (Figure 1A). Subsequently, we compared the expression levels of these palmitoylation regulating genes between TCGA-LGG and GTEx normal tissues. Among the 30 genes analyzed, 24 were significantly upregulated in LGG tissues compared to normal tissues, whereas 5 were significantly downregulated. ZDHHC13 expression exhibited no significant difference (Figure 1B).

We utilized the cBioPortal database to examine mutation frequencies of palmitoylation-related genes in LGG patients, revealing generally low mutation rates (Figure 1C). Among these, ABHD17A exhibited the highest mutation frequency at 4.1%. Furthermore, analysis of copy number variations (CNVs) indicated that such alterations were infrequent. Notable CNV amplifications were detected in ZDHHC9, ZDHHC12, ZDHHC15, and ABHD17A, while CNV deletions were more common in ZDHHC3, ZDHHC6, and ZDHHC19 (Figure 1D).

Subsequently, univariate Cox regression analysis was conducted using transcriptomic data paired with survival information from the CGGA_325, CGGA_693, and TCGA datasets. The results, visualized through forest plots (Figure 1E), identified nine genes (ZDHHC1, ZDHHC5, ZDHHC7, ZDHHC12, ZDHHC13, ZDHHC15, ZDHHC18, ZDHHC22, LYPLA1) that demonstrated statistically significant and consistent associations with prognosis across all three datasets.

### 3.2 Immune cell infltration analysis between palmitoylation clusters

**FIGURE 2:**
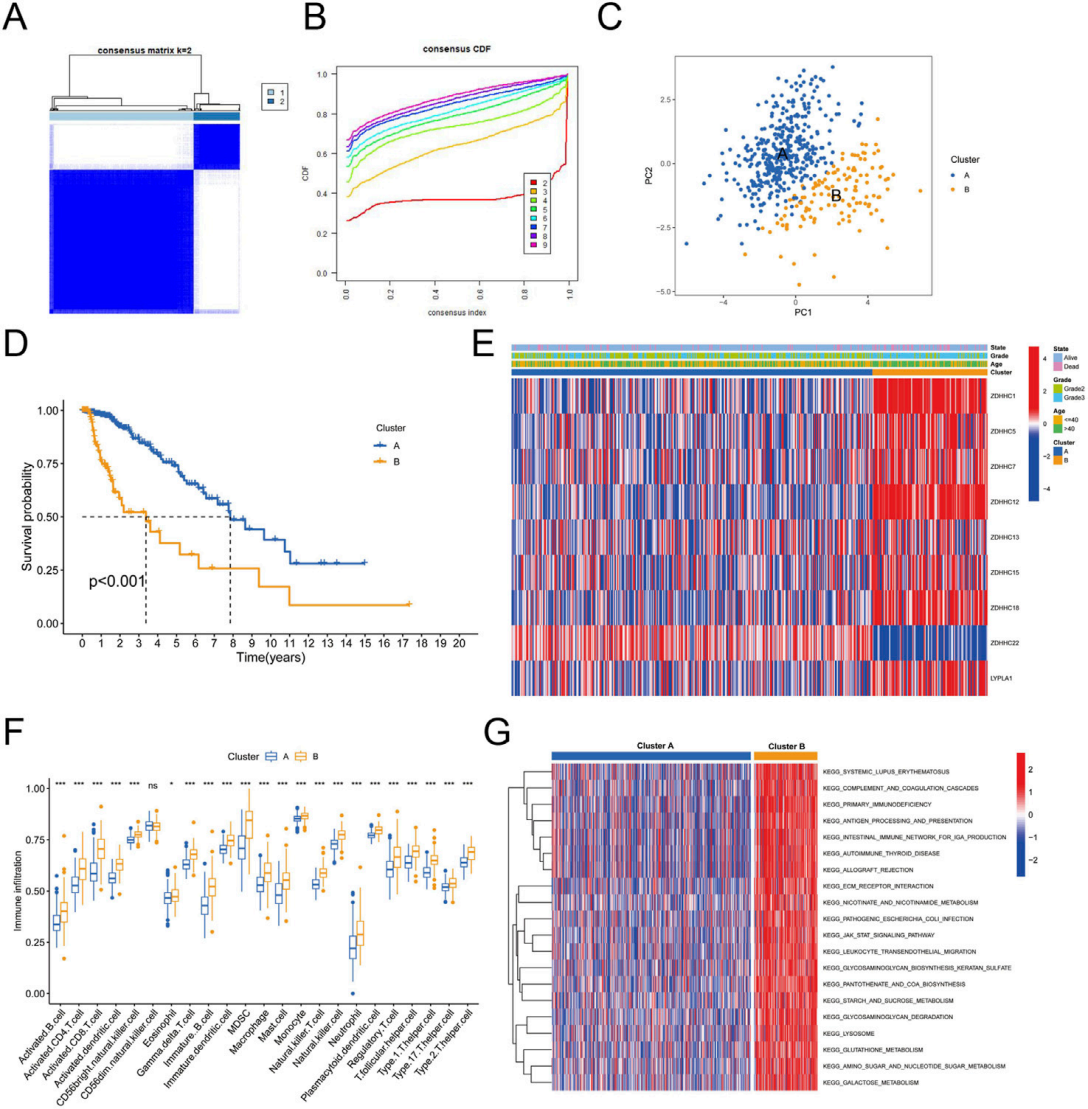
Immune Cell Infiltration Analysis Between Palmitoylation Clusters. **(A)** Heatmap of consensus clustering matrix for k = 2. **(B)** Consensus clustering stability for different k values (2–9). **(C)** Principal component analysis (PCA) of LGG patients based on Palmitoylation cluster classification. **(D)** Kaplan-Meier survival curves showing overall survival (OS) differences between Palmitoylation Cluster A and Palmitoylation Cluster B. **(E)** Heatmap displaying the distribution of nine palmitoylation regulating genes across different clusters, grades and ages. **(F)** Immune cell infiltration levels in Palmitoylation Cluster A and Palmitoylation Cluster B. **(G)** Gene Set Variation Analysis (GSVA) of biological pathways in Palmitoylation Cluster A and B.

To gain deeper insights into the biological processes and clinical significance of palmitoylation regulating genes, we performed consensus clustering analysis on samples from the TCGA databases. The samples were classified based on the expression levels of the nine palmitoylation regulating genes identified in the previous section. We tested different k values from 1 to 9 and found that k = 2 provided the best clustering stability (Figures 2A,B). Accordingly, we divided the LGG population into two clusters: Palmitoylation Cluster A and Palmitoylation Cluster B.

Principal component analysis (PCA) revealed that most LGG patients could be distinguished based on the Palmitoylation cluster classification (Figure 2C). Kaplan-Meier survival analysis revealed a significant disparity in overall survival (OS) between the two clusters. Patients in Palmitoylation Cluster A had a better prognosis compared to those in Palmitoylation Cluster B (Figure 2D). Heatmaps intuitively displayed the distribution of the nine palmitoylation-related genes across different clusters, grades and ages. Notably, a higher percentage of grade 3 LGG patients and elevated expression levels of most palmitoylation-related genes were observed in Palmitoylation Cluster B (Figure 2E).

Significant variations in immune cell infiltration were observed between the two clusters. Patients in Palmitoylation Cluster B showed higher levels of immune cell infiltration compared to Palmitoylation Cluster A, except for CD56 bright natural killer (NK) cells, which were less prevalent in Cluster B (Figure 2F).

To explore the biological functions differentiating the two clusters, we conducted Gene Set Variation Analysis (GSVA). The results indicated that pathways involving extracellular matrix-receptor interactions, leukocyte transendothelial migration, lysosomal activity, and tumor-related pathways (such as the JAK-STAT signaling pathway (Philips et al., 2022)) were notably enriched in Palmitoylation Cluster B (Figure 2G).

### 3.3 Identifcation of palmitoylation-related DEG subtypes in LGG

**FIGURE 3:**
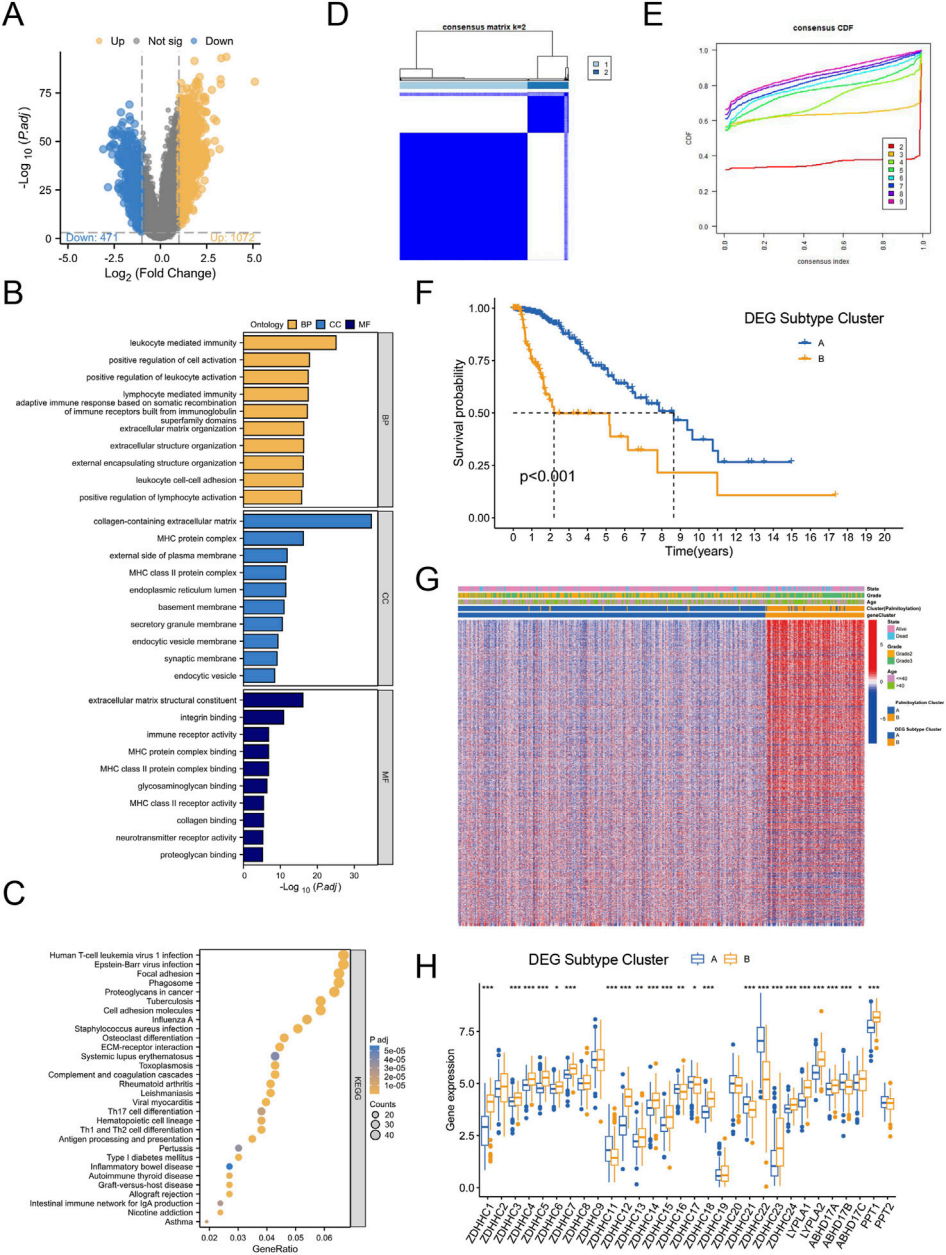
Identification of Palmitoylation-Related DEG Subtypes in LGG. **(A)** Volcano plot of differentially expressed genes (DEGs) between Palmitoylation Cluster A and Palmitoylation Cluster B. **(B)** GO functional enrichment analysis of DEGs. **(C)** KEGG pathway enrichment analysis of DEGs. **(D)** Heatmap of consensus clustering matrix for k = 2 based on DEGs. **(E)** Consensus clustering stability for different k values (2–9) based on DEGs. **(F)** Kaplan-Meier survival curves showing OS differences between DEG Subtype Cluster A and DEG Subtype Cluster B. **(G)** Heatmap displaying significant differences in tumor grade and age among LGG patients across Palmitoylation clusters and DEG subtypes. **(H)** Expression differences of palmitoylation regulating genes in different DEG subtypes.

Given the notable survival differences between the two Palmitoylation clusters, we investigated whether genetic variations might be a key factor. To explore this, we conducted a detailed analysis to identify potential gene alterations between Palmitoylation Cluster A and Cluster B, uncovering 1,543 differentially expressed genes (DEGs) (Figure 3A).

GO functional enrichment analysis revealed that these DEGs were primarily involved in biological processes (BP) such as leukocyte-mediated immune responses, lymphocyte-mediated immunity, and leukocyte cell-cell adhesion. In terms of cellular components (CC), the DEGs were enriched in major histocompatibility complex (MHC) protein complexes, MHC class II protein complexes, and extracellular matrix (ECM) containing collagen. For molecular functions (MF), the DEGs were associated with ECM structural components, immune receptor activity, and integrin binding (Figure 3B).

KEGG pathway enrichment analysis further showed that the DEGs were enriched in pathways related to proteoglycans in cancer, cell adhesion molecules, ECM-receptor interactions, antigen processing and presentation, Th17 cell differentiation, and Th1 and Th2 cell differentiation (Figure 3C). These findings suggest that genetic differences between the two clusters may contribute to the observed survival disparities.

To explore the clinical relevance of specific DEGs, we performed consensus clustering analysis, testing k values from 1 to 9. The optimal clustering stability was achieved at k = 2 (Figures 3D,E), leading to the identification of two Palmitoylation-related DEG subtypes: DEG Subtype Cluster A and DEG Subtype Cluster B. Kaplan-Meier survival analysis demonstrated that patients in DEG Subtype Cluster A had significantly better survival outcomes compared to those in DEG Subtype Cluster B (Figure 3F).

Heatmap analysis revealed notable differences in tumor grade and age among LGG patients across Palmitoylation clusters and DEG subtypes. Palmitoylation Cluster B and DEG Subtype Cluster B contained a higher proportion of patients with grade 3 or higher tumors (Figure 3G).

Further examination of palmitoylation-related gene expression across DEG subtypes (Figure 3H) showed that 17 genes (ZDHHC1, ZDHHC3, ZDHHC4, ZDHHC5, ZDHHC6, ZDHHC7, ZDHHC12, ZDHHC13, ZDHHC14, ZDHHC15, ZDHHC18, ZDHHC23, ZDHHC24, LYPLA1, LYPLA2, ABHD17C, PPT1) were significantly upregulated in DEG Subtype Cluster B. In contrast, six genes (ZDHHC11, ZDHHC16, ZDHHC17, ZDHHC21, ZDHHC22, ABHD17B) exhibited increased expression in DEG Subtype Cluster A (Figure 3H).

### 3.4 Construction and validation of the palmitoylation-related prognostic signature

**FIGURE 4:**
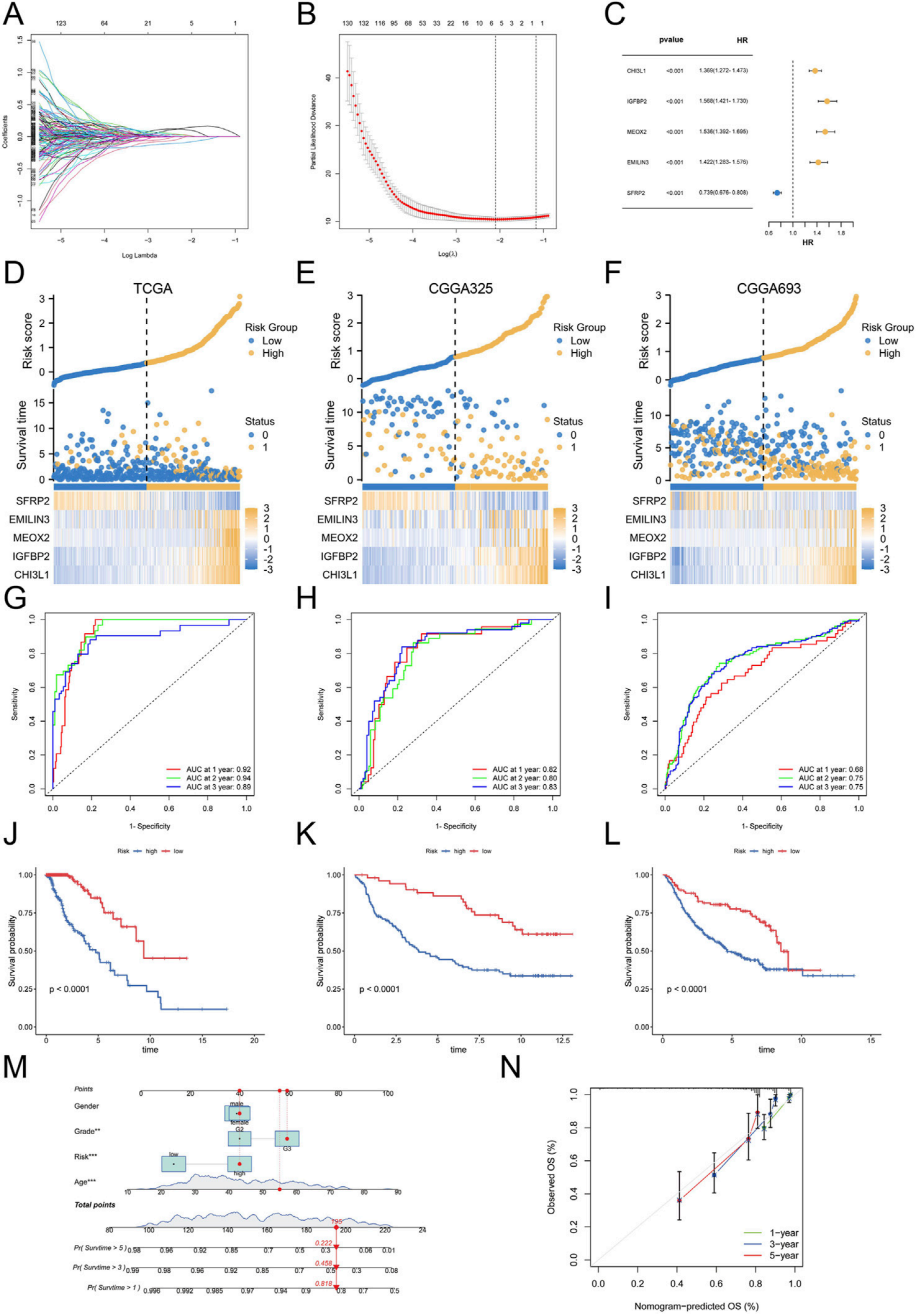
Construction and Validation of the Palmitoylation-Related Prognostic Signature. **(A)** LASSO regression analysis for gene selection. **(B)** Coefficients of selected genes in the LASSO model. **(C)** The coefficients of selected genes and their forest plot. **(D)** The heatmap for selected genes in the TCGA training set. **(E)** The heatmap for selected genes in the CGGA325 validation set. **(F)** The heatmap for selected genes in the CGGA693 validation set. **(G)** ROC curves for 1-, 2-, and 3-year survival in the TCGA training set. **(H)** ROC curves for 1-, 2-, and 3-year survival in the CGGA325 validation set. **(I)** ROC curves for 1-, 2-, and 3-year survival in the CGGA693 validation set. **(J)** Kaplan-Meier survival curves for OS in the TCGA cohort. **(K)** Kaplan-Meier survival curves for OS in the CGGA325 cohort. **(L)** Kaplan-Meier survival curves for OS in the CGGA693 cohort. **(M)** Nomogram for predicting 1-, 3-, and 5-year OS based on patient sex, grade, age, and risk score. **(N)** Calibration curves for the nomogram.


Considering the complexity and heterogeneity of each glioblastoma multiforme (GBM) patient, we constructed a palmitoylation-related prognostic signature to evaluate the prognosis of GBM patients. To build this prognostic signature, we identified 1,081 prognosis-related genes common to both the CGGA325 and CGGA693 cohorts through univariate Cox regression analysis. We used TCGA-LGG samples as the training set and CGGA325 and CGGA693 samples as external validation sets. LASSO analysis was then performed to further refine and select these genes (Figures 4A,B). Ultimately, we identified five palmitoylation-related genes and their corresponding coefficients (Figure 4C). For each LGG patient, the risk score was calculated using the following formula.

Risk  Score=CHI3L1×0.14+IGFBP2×0.11+MEOX2×0.02+EMILIN3×0.02++SFRP2×(−0.01).

Based on this score, patients in the TCGA cohort and the two external validation sets (CGGA325 and CGGA693) were stratified into high-risk and low-risk groups. The expression levels of the five selected genes, along with the risk scores and patient survival status, are reflected in Figures 4D–F for the TCGA, CGGA325, and CGGA693 datasets. Subsequently, we evaluated the prognostic performance of the palmitoylation-related signature using ROC analysis. In the training set, the AUC values for 1-, 2-, and 3-year survival were 0.92, 0.94, and 0.89, respectively (Figure 4G). This signature also demonstrated stable prognostic performance in the two external validation sets: for the CGGA325 cohort, the AUC values were 0.82, 0.80, and 0.83 for 1-, 2-, and 3-year survival (Figure 4H); for the CGGA693 cohort, the AUC values were 0.68, 0.75, and 0.75 (Figure 4I). These results indicate that our signature is a reliable predictor of LGG patient prognosis.

Survival status analysis revealed that high-risk patients experienced worse prognoses compared to low-risk patients across all three cohorts (Figures 4J–L). Kaplan-Meier survival analysis further validated that high-risk LGG patients had significantly reduced overall survival (OS) compared to low-risk patients (P < 0.0001).

To enhance clinical utility, we developed a nomogram incorporating patient sex, grade, age, and risk score to predict 1-, 3-, and 5-year OS (Figure 4M). The nomogram indicated that a total score of 195 corresponded to 1-, 3-, and 5-year survival rates of 81.8%, 45.8%, and 22.2%, respectively. Calibration curves demonstrated the nomogram’s accuracy in predicting patient OS (Figure 4N).

Collectively, these results suggest that the palmitoylation-related prognostic signature is a robust predictor of LGG prognosis, and the nomogram offers valuable insights for estimating patient survival outcomes.

### 3.5 The palmitoylation-related prognostic signature characterized by distinct immune infltration landscapes

**FIGURE 5:**
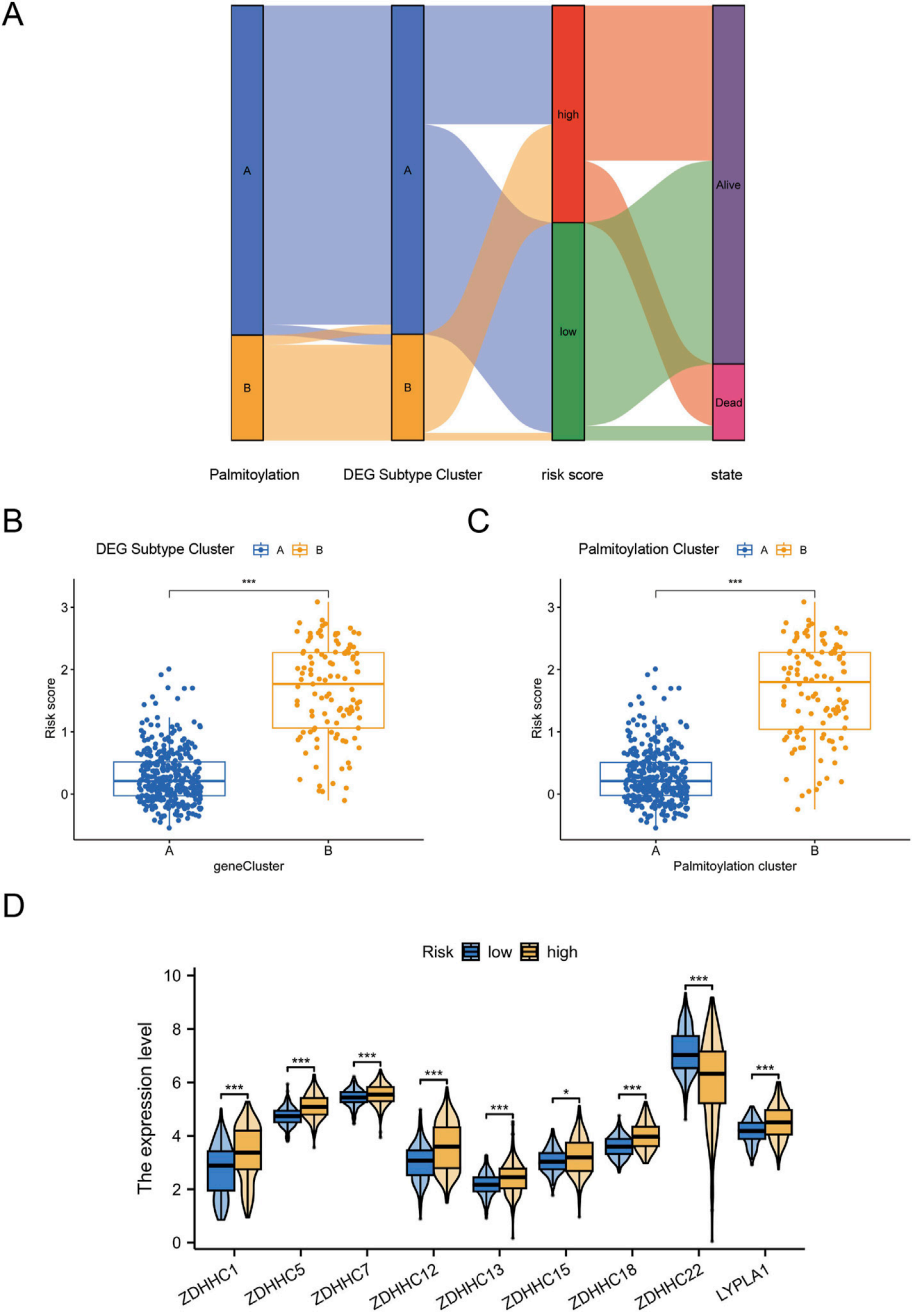
Distinct Immune Infiltration Landscapes Characterized by the Palmitoylation-Related Prognostic Signature. **(A)** Sankey diagram visualizing the distribution of LGG samples across different classification methods. **(B)** Box plot showing the significant difference in risk scores between DEG Subtype Cluster A and DEG Subtype Cluster B. **(C)** Box plot showing the significant difference in risk scores between Palmitoylation Cluster A and Palmitoylation Cluster B. **(D)** Expression levels of nine key palmitoylation regulating genes in different DEG subtypes.

After constructing the palmitoylation-related prognostic signature, we used Sankey diagrams to visualize the distribution of LGG samples across different classification methods. The results showed that most patients in Palmitoylation Cluster A were associated with DEG Subtype Cluster A, which had lower risk scores and better prognoses (Figure 5A). Conversely, the majority of patients in DEG Subtype Cluster B were associated with Palmitoylation Cluster B, exhibiting higher risk scores and poorer prognoses (Figure 5A). Quantitative analysis further supported these findings, showing that the risk scores in DEG Subtype Cluster B were significantly higher than those in DEG Subtype Cluster A (*P* < 0.001, Figure 5B). Additionally, the risk scores in Palmitoylation Cluster B were also significantly higher (*P* < 0.001, Figure 5C), indicating a potential association between palmitoylation-related genes and LGG prognosis.

We focused on the nine key palmitoylation regulating genes that showed significant differences in univariate analysis. Our analysis revealed that eight genes (ZDHHC1, ZDHHC5, ZDHHC7, ZDHHC12, ZDHHC13, ZDHHC15, ZDHHC18, LYPLA1) were significantly upregulated in DEG Subtype Cluster B. In contrast, ZDHHC22 was upregulated in DEG Subtype Cluster A (Figure 5D). These results suggest that these palmitoylation regulating genes may be associated with the poor prognosis observed in high-risk LGG patients.

### 3.6 Palmitoylation-related prognostic signature predicted the efficacy of immunotherapy

**FIGURE 6:**
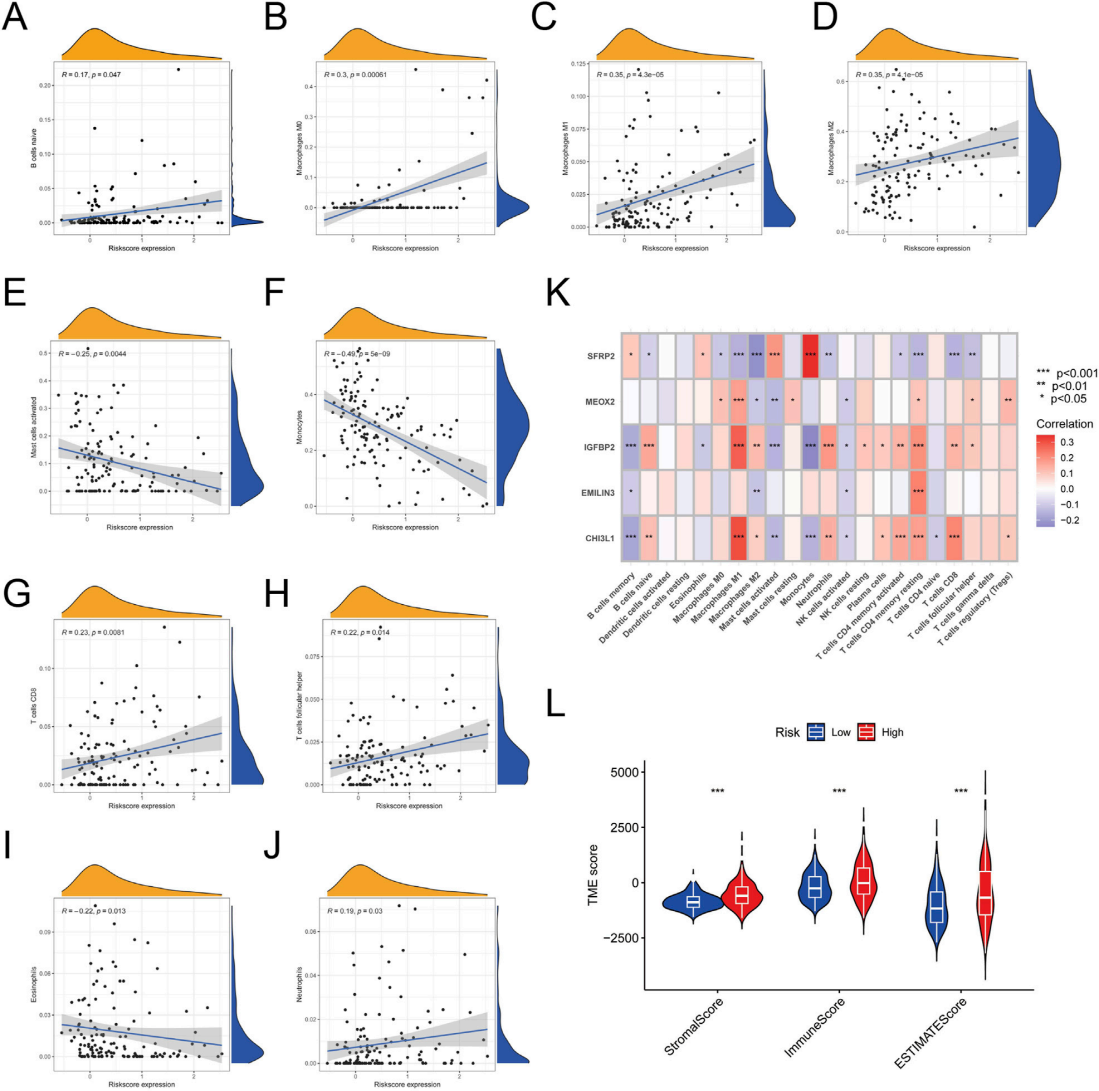
Correlation Between Risk Scores and Immune Cell Infiltration. **(A)** Correlation between risk scores and naive B cells. **(B)** Correlation between risk scores and M0 macrophages. **(C)** Correlation between risk scores and M1 macrophages. **(D)** Correlation between risk scores and M2 macrophages. **(E)** Correlation between risk scores and activated mast cells. **(F)** Correlation between risk scores and monocytes. **(G)** Correlation between risk scores and CD8 T cells. **(H)** Correlation between risk scores and follicular helper T cells. **(I)** Correlation between risk scores and eosinophils. **(J)** Correlation between risk scores and neutrophils. **(K)** Heatmap of correlations between the five feature genes and 22 types of immune cell infiltration. **(L)** Plot showing the significant difference in stromal, immune, and ESTIMATE scores between high-risk and low-risk groups.

To explore the role of the palmitoylation-related prognostic signature in LGG prognosis via the tumor microenvironment (TME), we assessed the relationship between risk scores and immune cell infiltration, revealing significant associations with multiple immune cell types. Specifically, naive B cells (R = 0.17, *P* = 0.047, Figure 6A), M0 macrophages (R = 0.30, *P* = 0.00061, Figure 6B), M1 macrophages (R = 0.35, *P* = 4.3e−5, Figure 6C), M2 macrophages (R = 0.35, *P* = 4.3e−5, Figure 6D), and neutrophils (R = 0.19, *P* = 0.03, Figure 6J) exhibited positive correlations with risk scores. In contrast, activated mast cells (R = −0.25, *P* = 0.0044, Figure 6E) and monocytes (R = −0.49, *P* = 5e−9, Figure 6F) showed significant negative correlations. Additionally, CD8 T cells (R = 0.23, *P* = 0.0081, Figure 6G) and follicular helper T cells (R = 0.22, *P* = 0.014, Figure 6H) also had positive correlations, while eosinophils (R = −0.22, P = 0.013, Figure 6I) showed a negative correlation.

A heatmap was constructed to illustrate the relationships between the five feature genes and 22 types of immune cell infiltration. The analysis showed that M1 macrophages, M2 macrophages, and CD4 memory activated T cells were significantly correlated with all five feature genes (Figure 6K). In contrast, activated dendritic cells, eosinophils, and gamma delta T cells showed no significant associations with these genes.

Additionally, IGFBP2 emerged as the gene most strongly linked to immune cell infiltration. Using the ESTIMATE algorithm, we assessed stromal, immune, and ESTIMATE scores in LGG samples, which were significantly higher in the high-risk group (Figure 6L). This indicates lower tumor purity and increased infiltration of immune and stromal cells in high-risk patients.

### 3.7 Palmitoylation-related prognostic signature predicted the efcacy of immunotherapy

**FIGURE 7:**
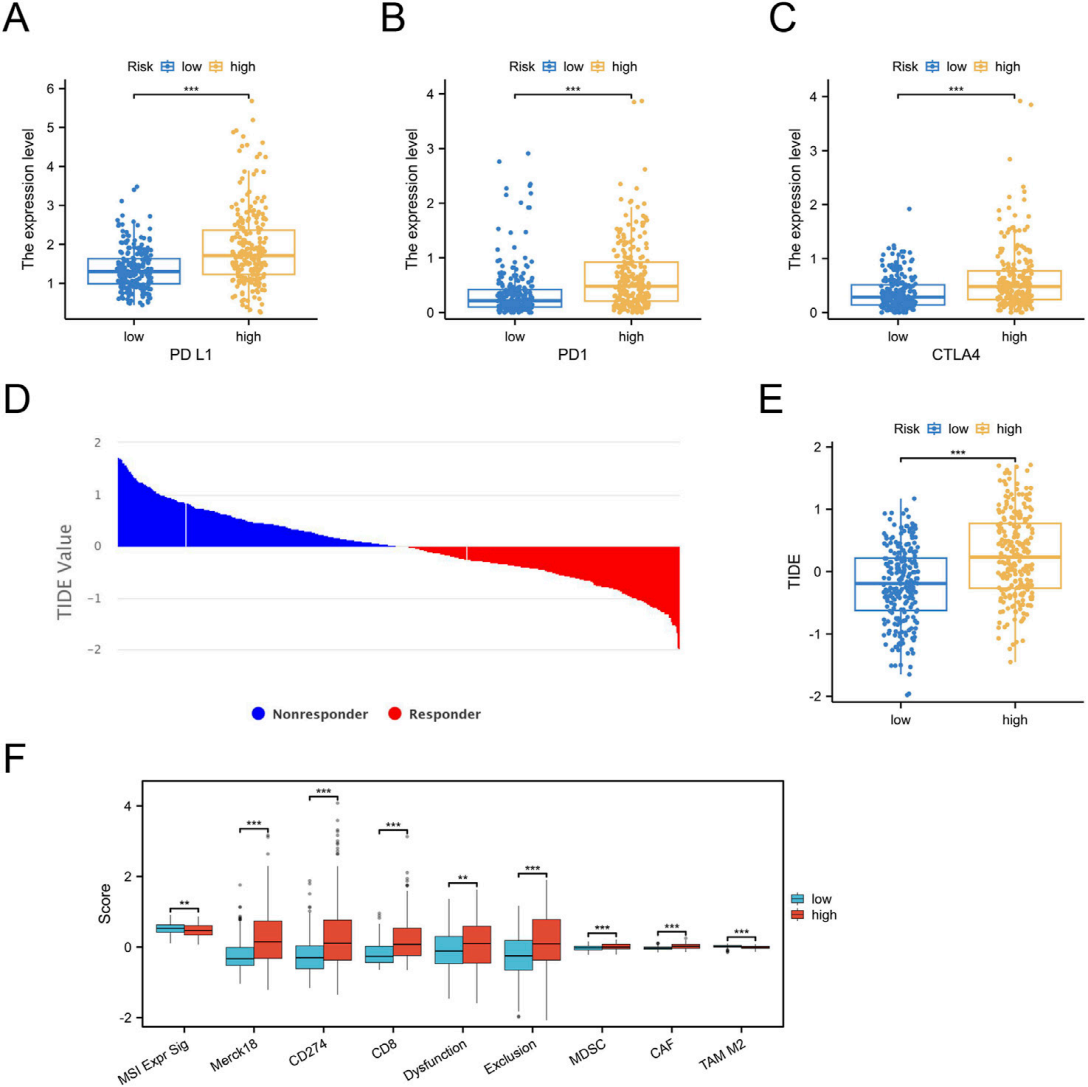
Prediction of Immunotherapy Efficacy by the Palmitoylation-Related Prognostic Signature. **(A)** PD-1 expression in high-risk versus low-risk groups. **(B)** PD-L1 expression in high-risk versus low-risk groups. **(C)** CTLA4 expression in high-risk versus low-risk groups. **(D,E)** TIDE scores demonstrating a significant difference between high-risk and low-risk groups. **(F)** Box plot illustrating the significant difference in TIDE scores for specific cell subsets between high-risk and low-risk groups.

Immune checkpoint inhibitors (ICIs) targeting PD-1, PD-L1, and CTLA-4 have shown significant therapeutic potential in various cancers. To investigate the connection between the palmitoylation-related prognostic signature and immunotherapy, we compared the expression levels of these immune checkpoints between high-risk and low-risk groups. The analysis revealed that high-risk patients exhibited significantly elevated levels of PD-1 (P < 0.001, Figure 7A), PD-L1 (P < 0.001, Figure 7B), and CTLA4 (P < 0.001, Figure 7C) compared to low-risk patients.

To evaluate the implications for immunotherapy efficacy, we employed the Tumor Immune Dysfunction and Exclusion (TIDE) algorithm to classify the entire LGG cohort. The results indicated that the high-risk group had significantly higher TIDE scores (Figures 7D,E).

We also assessed TIDE scores for specific cell subsets, including CD8 T cells and myeloid-derived suppressor cells (MDSCs), in both high-risk and low-risk groups (Figure 7F). The high-risk group displayed higher TIDE scores for these subsets, providing partial insight into the observed increase in immune infiltration despite poorer prognosis in this group.

### 3.8 Functional validation of IGFBP2 knockdown in U251 cells

To further enhance the credibility of the prognostic signature, we conducted *in vitro* functional validation experiments. The CHI3L1-encoded protein drives tumor growth, migration, and invasion in gliomas, with its expression levels correlating with tumor malignancy and adverse prognosis. IGFBP2, a component of the IGF (insulin-like growth factor) axis, regulates cell proliferation, differentiation, and apoptotic processes. In gliomas, IGFBP2 expression is linked to tumor growth and malignancy. MEOX2, which encodes a protein involved in glioma development and angiogenesis, has expression levels that may correlate with tumor invasiveness and vascularization. EMILIN3 (Elastin-like polypeptide 3) is primarily studied for its interactions with the tumor microenvironment and its effects on glioma cell behavior. SFRP2, an inhibitor of the Wnt signaling pathway, affects glioma cell proliferation, migration, and survival through Wnt signal regulation. Correlation analyses revealed a central role of IGFBP2 in immune-mesenchymal interactions, supported by its substantial weight in prognostic models and its potential novel mechanism in palmitoylation-mediated immune evasion. While acknowledging the well-documented importance of other signature genes through extensive literature evidence, we prioritized IGFBP2 for functional validation based on these compelling findings.

**FIGURE 8:**
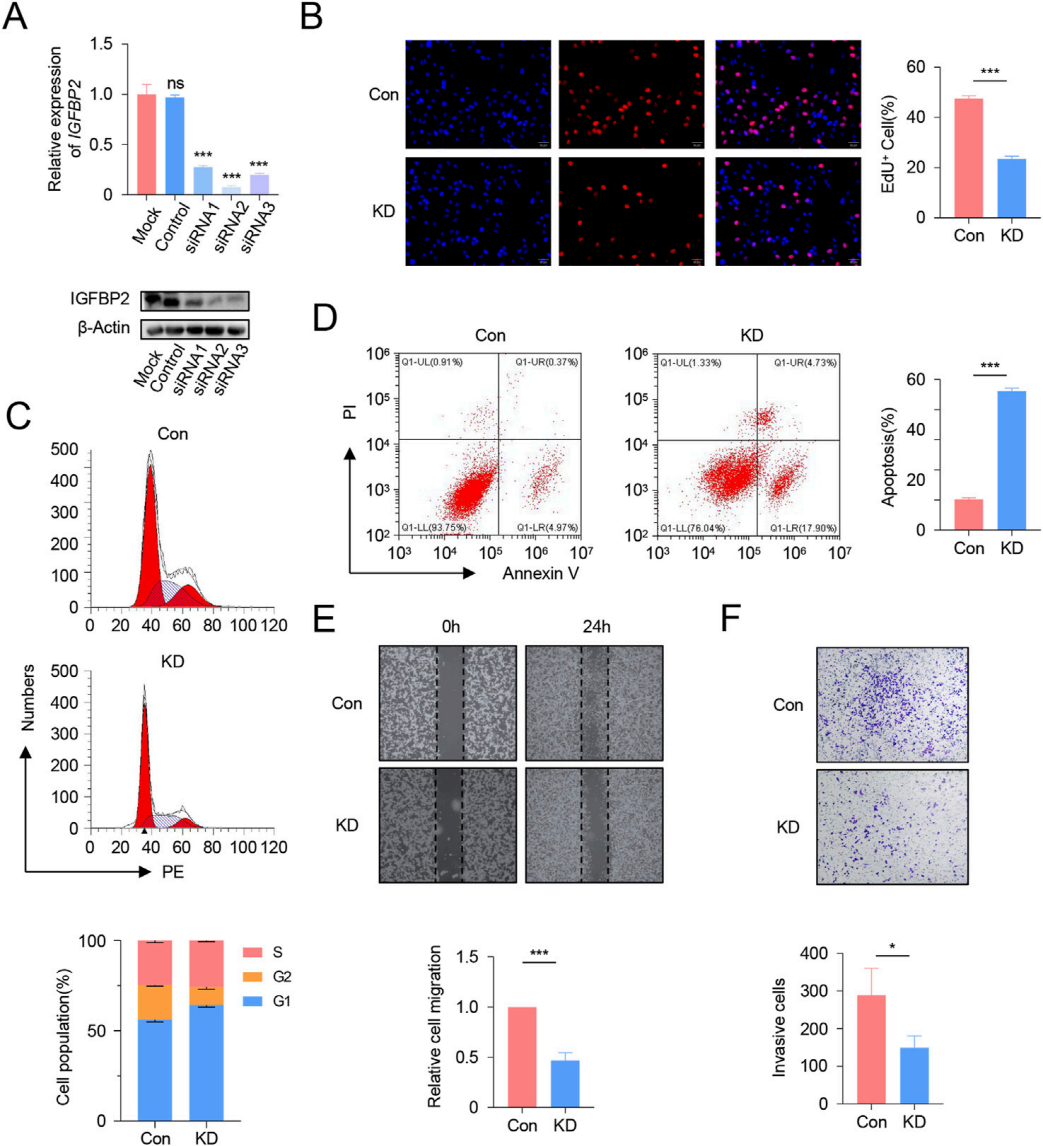
Functional Validation of IGFBP2 Knockdown in U251 Cells. **(A)** qRT-PCR and Western blot analysis of IGFBP2 expression in U251 cells following siRNA transfection. **(B)** EdU incorporation assay demonstrating the impact of IGFBP2 knockdown on cell proliferation. **(C)** Flow cytometry analysis of cell cycle distribution post-IGFBP2 knockdown. **(D)** Flow cytometry analysis of apoptosis rates post-IGFBP2 knockdown. **(E)** Wound healing assay illustrating the effect of IGFBP2 knockdown on cell migration. **(F)** Transwell invasion assay revealing the effect of IGFBP2 knockdown on cell invasion.

Initial screening via qRT-PCR and Western blot (WB) identified siRNA2 as the most effective interference fragment for IGFBP2 knockdown, validated at both mRNA and protein levels (Figure 8A). Subsequent functional analyses using siRNA2 revealed that IGFBP2 silencing significantly reduced cellular proliferation, as evidenced by decreased EdU incorporation rates (*P* < 0.001, Figure 8B). Flow cytometry-based cell cycle analysis demonstrated a marked increase in the proportion of cells arrested in the G1 phase following IGFBP2 knockdown (Figure 8C). Concurrently, apoptosis assays revealed a significant elevation in apoptotic rates (*P* < 0.001), including both early and late apoptotic populations (Figure 8D). Furthermore, IGFBP2 depletion attenuated cellular migratory and invasive capacities, as confirmed by wound healing and transwell invasion assays (Figures 8E,F).

In summary, our findings indicate that interference with IGFBP2 expression can effectively inhibit the proliferation and migration of U251 cells.

## 4 Discussion

This study offers a detailed examination of palmitoylation-related genes in LGG, highlighting their significant impact on tumor progression, immune microenvironment regulation, and clinical outcomes. While palmitoylation genes are distributed across diverse chromosomal loci without evidence of cancer-specific clustering (Figure 1A), their functional convergence in lipid modification suggests transcriptional or epigenetic regulation may coordinate their activity more than genomic proximity. Through the integration of multi-omics data and functional validation, we identified unique palmitoylation clusters, established a reliable prognostic signature, and explored potential mechanisms connecting palmitoylation to immunotherapy effectiveness.

Our identification of two palmitoylation clusters (Cluster A and B) in LGG highlights the heterogeneity of palmitoylation dynamics in LGG biology. The upregulation of 24 palmitoylation regulating genes in tumor tissues (e.g., ZDHHC12, LYPLA1) compared to normal tissues suggests a tumor-promoting role for palmitoylation in LGG. Notably, the higher prevalence of grade 3 tumors and poorer survival in Cluster B correlates with enriched pathways such as extracellular matrix-receptor interactions and JAK-STAT signaling, which are known drivers of glioma invasion and treatment resistance (Fujii et al., 2023; Kwiatkowska and Symons, 2020). These findings align with studies demonstrating that palmitoylation regulates membrane localization of oncoproteins, including Src-family kinases and Ras GTPases, which are critical for tumor progression (Mareel and Leroy, 2003). However, the low mutation frequency of palmitoylation genes (e.g., ABHD17A at 4.1%) implies that their dysregulation may primarily occur at the transcriptional or post-translational level, warranting further mechanistic investigations.

The pronounced immune infiltration in Cluster B, characterized by elevated M1/M2 macrophages and CD8^+^ T cells, paradoxically associates with worse prognosis. This “immune-excluded” phenotype, marked by high stromal and ESTIMATE scores, mirrors observations in other cancers where dense stromal barriers limit immune effector cell function despite abundant infiltration (Shi et al., 2021). The negative correlation between monocytes and risk scores further underscores the complexity of immune interactions in glioma, as monocytes can differentiate into immunosuppressive tumor-associated macrophages (Mulder et al., 2021). The poorer prognosis may stem from an immunosuppressive microenvironment. For instance, M2 macrophages and regulatory T cells (Tregs) could suppress effector T cell function via IL-10 and TGF-β secretion. The enrichment of antigen presentation pathways (e.g., MHC class II complexes) in Cluster B suggests that palmitoylation may modulate immune evasion by altering antigen processing—a hypothesis supported by the upregulation of PD-1, PD-L1, and CTLA4 in high-risk patients. ZDHHC enzymes may enhance PD-L1 membrane localization and stability via palmitoylation, as shown for other immune checkpoints (Yao et al., 2019). These findings position palmitoylation as a potential mediator of the immunosuppressive microenvironment, offering a rationale for combining palmitoylation inhibitors with immune checkpoint blockade.

The five-gene palmitoylation-related signature (CHI3L1, IGFBP2, MEOX2, EMILIN3, SFRP2) demonstrates robust prognostic accuracy across independent cohorts (AUC: 0.68–0.94). Its predictive power for survival aligns with the roles of these genes in glioma biology: CHI3L1 promotes invasiveness via NF-κB activation (Xu et al., 2023), while IGFBP2 enhances angiogenesis through IGF signaling (Kharkova et al., 2014). The inverse association of SFRP2 (a Wnt inhibitor) with risk scores further supports Wnt pathway activation as a hallmark of aggressive gliomas (Majchrzak-Celińska et al., 2016). Importantly, the integration of this signature into a nomogram incorporating age, grade, and sex provides a clinically actionable tool for stratifying patients into risk-adapted therapeutic strategies. While the model demonstrated high accuracy in the training cohort (AUC 0.94), its performance in external validation (AUC 0.68–0.75) highlights potential overfitting or cohort heterogeneity. Future studies should validate this signature in prospective cohorts and integrate clinical parameters (e.g., IDH mutation status) to enhance generalizability.

Functional experiments conducted in U251 cell lines demonstrated that silencing IGFBP2 significantly inhibits proliferation, migration, and invasion, reinforcing its role as a critical driver of glioma malignancy. The strong association between IGFBP2 expression and immune infiltration, such as macrophages and neutrophils, suggests it may act as a key interface between palmitoylation and immune modulation. These findings build on previous research linking IGFBP2 to PI3K/Akt pathway activation (Qian et al., 2023) and underscore its potential as a therapeutic target. Future investigations should explore whether IGFBP2 inhibition can enhance the efficacy of existing therapies, such as temozolomide or immunotherapies, to address treatment resistance.

While our findings provide novel insights into palmitoylation-mediated mechanisms in LGG, several limitations warrant discussion. First, the retrospective design of the TCGA and CGGA datasets inherently limits causal inference between palmitoylation alterations and clinical outcomes. Second, while we identified associations between specific palmitoylation genes (e.g., ZDHHC22 in Cluster A) and immune evasion, the exact mechanistic underpinnings remain unresolved (Du et al., 2021). For instance, whether ZDHHC22 directly modulates immune checkpoint trafficking or antigen presentation pathways constitutes a critical knowledge gap requiring targeted investigation. Third, while the prognostic signature demonstrated robust performance in independent cohorts, external validation in larger multi-institutional cohorts with standardized clinical annotations will strengthen its translational utility.

Although functional validation prioritized IGFBP2 due to its centrality in immune-stromal interactions, the roles of other signature genes (e.g., CHI3L1, SFRP2) in palmitoylation-mediated immune evasion remain unexplored. Systematic interrogation of their palmitoylation-dependent molecular networks (e.g., Wnt/NF-κB crosstalk for SFRP2) represents a critical next step. Finally, the metabolic consequences of palmitoylation dysregulation in LGG remain unaddressed. Emerging evidence implicates palmitoylation in regulating nutrient transporters (e.g., GLUT1) and metabolic enzymes (e.g., ACLY), suggesting its potential role in rewiring glioma metabolism—a dimension meriting dedicated metabolomic studies.

Spatial resolution of palmitoylation dynamics also remains elusive. Current bulk transcriptomic analyses cannot discern whether observed changes originate from tumor cells or infiltrating immune populations. Future work integrating spatial transcriptomics and single-cell RNA sequencing could map palmitoylation-related gene expression gradients within tumor microenvironments, while preclinical models (e.g., patient-derived organoids) may validate therapeutic candidates targeting this pathway.

## 5 Conclusion

In conclusion, this study highlights palmitoylation as a key regulator of LGG progression and immune evasion. The prognostic signature and nomogram developed here provide practical tools for risk assessment, while the functional significance of IGFBP2 emphasizes its potential as a therapeutic target. These findings lay the groundwork for personalized therapies aimed at modulating palmitoylation pathways to enhance outcomes for LGG patients.

## Data Availability

The original contributions presented in the study are included in the article/supplementary material, further inquiries can be directed to the corresponding authors.
